# Synthesis, In Silico Log*p* Study, and In Vitro Analgesic Activity of Analogs of Tetrapeptide FELL

**DOI:** 10.3390/ph16081183

**Published:** 2023-08-21

**Authors:** Boryana Borisova, Hristina Nocheva, Stéphane Gérard, Marie Laronze-Cochard, Stefan Dobrev, Silvia Angelova, Stoyko Petrin, Dancho Danalev

**Affiliations:** 1 Biotechnology Department, University of Chemical Technology and Metallurgy, 8 Kliment Ohridski Blvd., 1756 Sofia, Bulgaria; boriana.borisowa@gmail.com (B.B.); stpetrin@uctm.edu (S.P.); 2 Department of Physiology and Pathophysiology, Faculty of Medicine, Medical University of Sofia, Sv. Georgi Sofiyski Blvd. 1, 1431 Sofia, Bulgaria; hndimitrova@medfac.mu-sofia.bg; 3 Institut de Chimie Moléculaire de Reims (ICMR)-UMR CNRS 7312, Université de Reims Champagne-Ardenne, UFR Pharmacie, 51 Rue Cognacq-Jay, 51096 Reims, France; stephane.gerard@univ-reims.fr (S.G.); marie.cochard@univ-reims.fr (M.L.-C.); 4 Institute of Optical Materials and Technologies “Acad. J. Malinowski”, Bulgarian Academy of Sciences, Acad. G. Bonchev Str., bl. 109, 1113 Sofia, Bulgaria; sdobrev@iom.bas.bg (S.D.); sea@iomt.bas.bg (S.A.)

**Keywords:** FELL analogs, analgesic activity, peptide synthesis, paw-pressure test

## Abstract

Background: The inflammatory process represents a specific response of the organism’s immune system. More often, it is related to the rising pain in the affected area. Independently of its origin, pain represents a complex and multidimensional acute or chronic subjective unpleasant perception. Currently, medical doctors prescribe various analgesics for pain treatment, but unfortunately, many of them have adverse effects or are not strong enough to suppress the pain. Thus, the search for new pain-relieving medical drugs continues. Methods: New tetrapeptide analogs of FELL with a generaanalgesic-Glu-X^3^-X^4^-Z, where X = Nle, Ile, or Val and Z = NH_2_ or COOH, containing different hydrophobic amino acids at positions 3 and 4, were synthesized by means of standard solid-phase peptide synthesis using the Fmoc/OtBu strategy in order to study the influence of structure and hydrophobicity on the analgesic activity. The purity of all compounds was monitored by HPLC, and their structures were proven by ESI-MS. Log*p* values (partition coefficient in octanol/water) for FELL analogs were calculated. Analgesic activity was examined by the Paw-pressure test (Randall-Selitto test). Results: The obtained results reveal that Leu is the best choice as a hydrophobic amino acid in the FELL structure. Conclusions: The best analgesic activity is found in the parent compound FELL and its C-terminal amide analog.

## 1. Introduction

Inflammation represents a natural defense mechanism in organisms where the immune system is integrated. It is triggered by different pathogens (physical, chemical, biological, dietary agents, or even oxygen deprivation) but mediated by a typical chemical signaling pathway, initiating the acute phase reaction. The latter indeed leads to specific tissue damage but also initiates the healing process [1,2]. It can be stated that inflammation is at the root of many human pathologies. Uncontrolled acute inflammation, as well as inefficiently or untimely treated ones, may progress to a chronic phase with additional tissue damage, predisposing eventually even to autoimmune or malignant processes. The pathogenesis of inflammation follows a typical pathway, including a defined number of mediators, such as histamine, prostaglandins, leucotrienes, cytokines, interleukins, and many others. There are also typical local manifestations like pain, swelling, heat, redness, and loss of function. In addition, systemic features of the body’s inflammatory response also exist [2,3,4]. They depend on the biological effects of the mediators released mostly by immune cells, e.g., neutrophils, monocytes, etc., but also by endothelial cells and platelets [3,4]. Thus, mediators contribute both to the defensive (phagocytosis of pathogens) and healing (tissue regeneration) purposes of inflammation as well as its clinical manifestations (pain, oedema, shortness of breath, etc.).

According to the 2020 revision of the International Association for the Study of Pain (IASP), the currently accepted definition of pain is “an unpleasant sensory and emotional experience associated with, or resembling that associated with, actual or potential tissue damage” [5]. Sensory-discriminative, affective-emotional, and cognitive-evaluative components “build up” the mechanism of pain perception, resulting from dynamic interactions of multiple central and peripheral neural processes [6,7]. Although acute pain protects us from predictable harm, recurrent acute episodes and, especially, chronic pain deteriorate the quality of life, leading to discomfort and disability [8].

Various analgesics are used for pain treatment and management, but such exogenous substances would have no effect unless they could bind to appropriate receptors. The involvement of several receptors in pain perception has been described.

Opioid receptors are widely distributed throughout the brain and participate in many mechanisms regulating central nervous system (CNS) functions, including pain processing, reinforcement, euphoria, sedation, dysphoria, miosis, addiction, truncal rigidity, hedonia, aversion, and nausea [9,10,11,12,13]. Several types of opioid receptors have been described, differing by their distribution in the nervous system [14] as well as by their endogenous ligands. μ-opiod receptors (MORs) recognize endorphins; δ-opiod receptors bind to enkephalins; and κ-opiod receptors and the opioid receptor like-1 are targeted by dynorphins [15,16]. Clinically, several MOR agonists named opioids, such as oxycodone, hydrocodone, morphine, and fentanyl, are useful in severe pain syndrome management. The benefits from opioids are often counteracted by many side effects (i.e., emesis, constipation, and sedation) [17], as well as by the increased rate of overdose-related respiratory depression and deaths caused by the illicit use of μ-opioid receptor agonist-containing substances [18].

Cannabinoid receptors type 1 (CB1Rs) are another type of receptor with a prominent role in nociception [19], as are receptors for serotonin [20], oxytocin, vasopressin [21], adenosine [22], GABA [23], etc. 

Thus, there are multiple possible mechanisms for analgesia, which are very complex and depend on the various neurological pathways involved in nociception [24]. 

Although many substances, such as non-steroidal anti-inflammatory drugs, cyclooxygenase inhibitors, opioids, corticosteroids, etc. [8,24,25], are known to possess analgesic properties, their use is hampered by side effects, such as respiratory depression, nausea, clouding of consciousness, constipation, addiction, and tolerance [26]. Thus, the development of opioid drugs free of such effects is a major goal in “pain killer” research. Many research groups focus their efforts on developing novel analgesic agents with better selectivity or greater effect at lower doses. 

In this context, the development of peptides as therapeutic agents is of great interest worldwide due to their small size, natural mechanism for elimination, low or lack of secondary effects, etc. [27]. Three peptides derived from human calcium-binding protein spermatid 1 (CABS1) FELL, TDIFELL, and TDIFELLK, were investigated by Laurent et al. [28]. As a result of this study, they suggest that the FELL core motif is a modification of the sequence Phe-Glu-Gly (FEG), displaying anti-inflammatory properties. In addition, they reveal that replacement of the C-terminal COOH function with the amide one leads to new structures active against endotoxic reactions [29]. Taking into account all previous studies on the tetrapeptide Phe^1^-Glu^2^-Leu^3^-Leu^4^-OH (FELL, code **BB11**) [28,29,30,31,32], herein we report the synthesis and study of potential properties of FELL’s structural analogs (formally named in the study with the codes **BB1-4**). The design of new molecules includes the replacement of Leu by its structural hydrophobic analogs nor-Leu (Nle), Ile, and Val to obtain peptides with the general structure Phe-Glu-X-X-Z, where X = Nle, Ile, or Val. In addition, influenced by the positive results reported by Laurent et al. [28], the C-terminal COOH function was transformed into the C(=O)NH_2_ group in four newly synthesized tetrapeptides (Z = C(=O)NH_2_ or COOH in the parent FELL compound). The design of new structures aims at elucidating the role of different hydrophobic amino acids in the 3rd and 4th positions of the parent peptide on their analgesic activity.

## 2. Results

### 2.1. Synthesis and Characterization of Target Peptides

**Figure 1:**
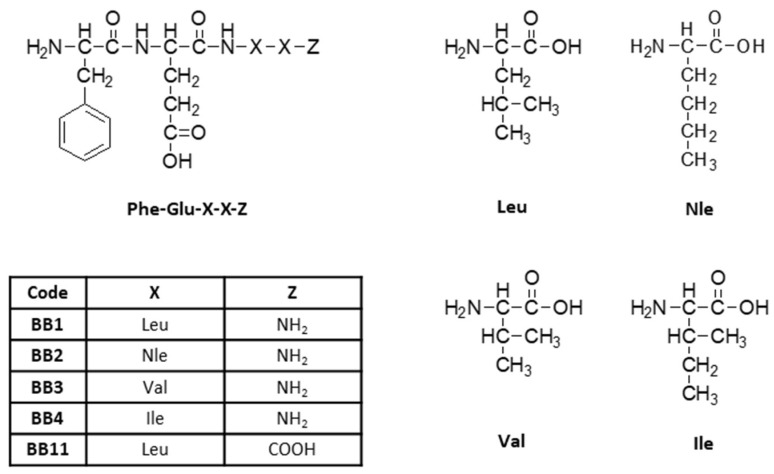
General structure of newly synthesized tetrapeptides.

A series of analogs of the tetrapeptide FELL as N-terminus amide with general structure Phe-Glu-X-X-Z (Figure 1), where X = Leu, Nle, Ile, or Val, and Z = C(=O)NH_2_ or COOH (in the parent compound), were synthesized and analyzed.

The analytical data for the synthesized peptides are summarized in Table 1. 

### 2.2. Evaluation of the Analgesic Properties of the New FELL Analogs

**Figure 2:**
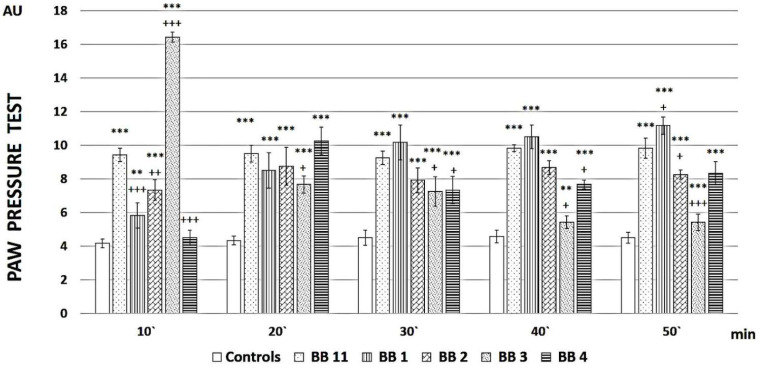
PPT of newly synthesized peptide analogs. Measurements have been performed every 10 min, starting from the 10th minute after peptide administration until the 50th minute. The results are presented in arbitrary units (AU) as mean values ± S.E.M. *** *p* < 0.001, ** *p* < 0.01 vs. controls; +++ *p* < 0.001, ++ *p* < 0.01, + *p* < 0.05 vs. **BB11**.

The analgesic properties of all four newly synthesized analogs vs. the parent compound have been estimated by means of the Paw Pressure Thresholds (PPT) test, differing in their duration and magnitude (Figure 2).

**BB1** showed a gradually increasing analgesic activity over time, which at the end of the reported period statistically reliably (F = 5.38217, *p* = 0.042776) exceeded that of the parent compound **BB11**. 

**BB3** had the highest but short-lasting analgesic activity, which at the 10th minute exceeded that of **BB11** (F = 92.35602, *p* < 0.00001), while **BB2** and **BB4** caused fluctuating analgesia that did not reach the values of the parent compound.

**Figure 3:**
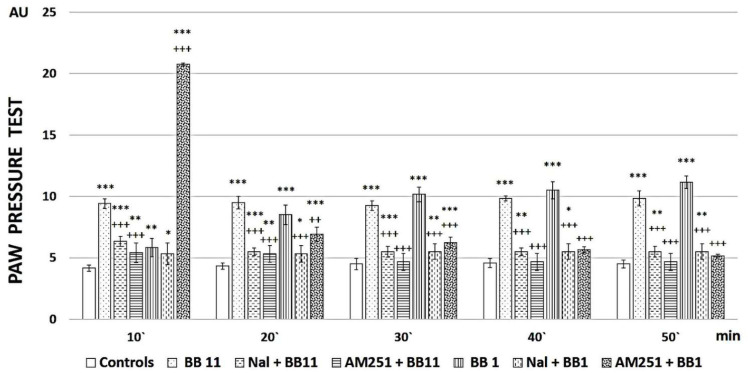
PPT of newly synthesized peptide analogs after Naloxone (Nal) or AM251 pre-treatment. Measurements have been performed every 10 min starting from the 10th minute after peptide administration until the 50th minute. The results are presented in arbitrary units (AU) as mean values ± S.E.M. *** *p* < 0.001, ** *p* < 0.01, * *p* < 0.05 vs. controls; Nal+BB11 and AM251+BB11 have been compared to BB11 +++ *p* < 0.001; Nal+BB1 and AM251+BB1 have been compared to BB1 +++ *p* < 0.001, ++ *p* < 0.01.

The obtained results reveal that **BB1** is the most promising newly synthesized compound, so its analgesic activity was additionally evaluated (as well as that of **BB11**) after pretreatment with naloxone to establish the involvement of opioid receptors in the reported effects or AM251 to establish the involvement of cannabinoid receptors in the reported effects. Both substances showed low analgesic activity after both types of pretreatments, indicating that both opioid and cannabinoid receptors are involved in the analgesic effects (Figure 3).

### 2.3. Prediction of the Logarithm of the N-Octanol Water Partition Coefficient (Logp)

**Figure 4:**
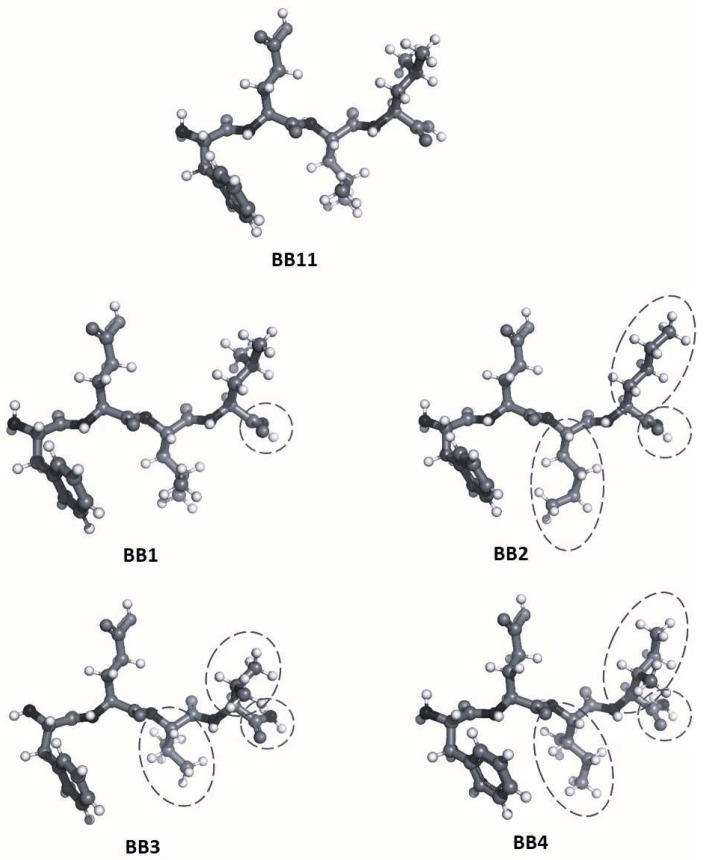
Structures of the natural BB11 peptide and its analogs. Differences with the parent structure (**BB11**) are circled.

The 3D structures of FELL analogs are shown in Figure 4, and the calculated log*p* values for these structures are given in Table 2. The results shown in Table 2 present the natural **BB11** as the most lipophilic one (log*p* = 1.54). Next is a group of three analogs—**BB1**, **BB2**, and **BB4**—all retaining the lipophilic nature, although to a lesser extent (log*p* values between 0.67, 0.80, and 0.82 for **BB1**, **BB2**, **and BB4**, respectively). Last at ambiphilic log*p* (close to 0, meaning equally dissolved in water and *n*-octanol) is the **BB3** compound. Of the tested molecules, **BB11** (the parent compound) would have the best distribution across biological tissues.

## 3. Discussion

### 3.1. Synthesis and Characterization of Target Peptides

**Figure 5:**
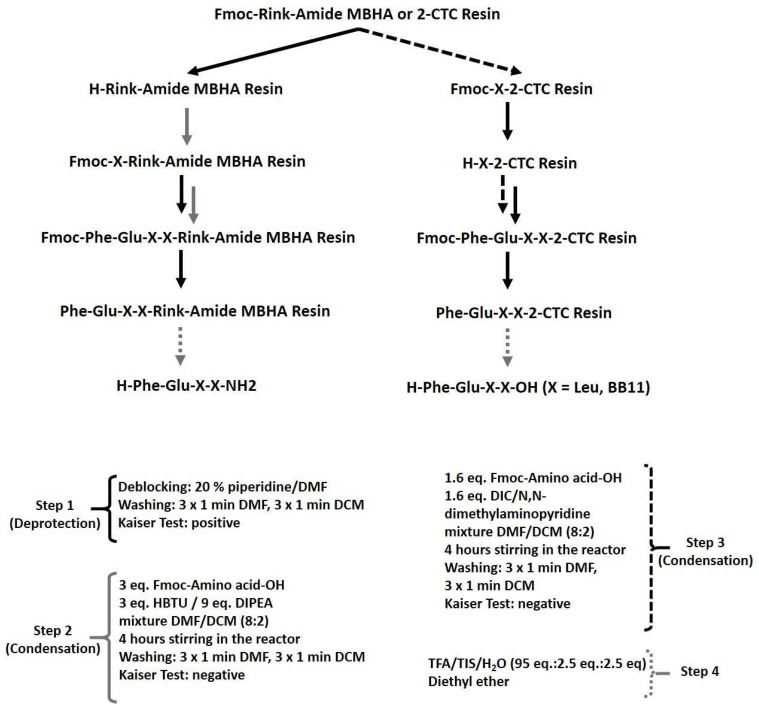
Schematic representation of the general procedure for targeted peptide synthesis.

Four target analogues, C-terminal amides, and the parent compound Phe-Glu-Leu-Leu-COOH (FELL) with C-terminal carboxylic function were synthesized according to the general scheme presented in Figure 5. 

The condensation steps were realized with HBTU/DIPEA or DIC/DMAP as condensation systems. Peptides were cleaved from their corresponding resin (Rink-amide MBHA or 2-CTC) by using a mixture of TFA/TIS/distilled water, as described in the Materials and Methods section. The analytical data for the synthesized peptides are shown in Table 1. The chromatographic purity of all synthesized compounds is greater than 95%. The target peptides could be synthesized both by peptide synthesis in solution and by SPPS. The Fmoc/O*t*-Bu SPPS became a standard technique for routine peptide synthesis due to its many advantages over solution synthesis, such as easy and fast procedures for condensation and deprotection, relatively cheap commercially available reagents, a lack of need to isolate, purify, and prove the structure of intermediates, high purity of final products, and their easy isolation. 

Laurent et al. described that all three human CABS1-derived peptides (FELL, TDIFELL, and TDIFELLK) possess anti-inflammatory activity as they reduce the total number of white blood cells (WBC), especially neutrophils and macrophages, and their accumulation in the bronchoalveolar fluid (BALF) in the lungs (FELL by 51%, TDIFELL by 54%, and TDIFELLK by 57%) [28]. The research of Omoigui [33] reveals that neutrophils could also be found in pain syndromes associated with arthritis, back and head pain, etc. Since these three peptides do not show significant differences in their influence on reducing the WBC rate in BALF, this work is focused on the study of the simplest amino acid sequence, FELL. Taking into account that inflammation in many cases is related to pain as well as that FELL is a structurally modified FEG/feG peptide that has different biological activities, the opioid activity of newly synthesized FELL analogs is presumed [29,31,32]. 

Morris et al. [29] and Metwally et al. [32] revealed in their studies that acetylation of the N-terminus (Ac-FEG) causes a loss of biological activity; aromaticity of the amino acid in the first position is essential for the activity, and the extension of carboxyl function in the second residue is important for the activity. Thus, Glu in this position is preferred instead of Asp.

Metwally et al. concluded that C-terminal amidation of the FEG molecule led to the loss of anti-inflammatory activity, but considering that the analgesic activity of targeted compounds is a main target of the study, four amides and tetrapeptide derivatives of FELL were synthesized. In the new structures with a general formula H-Phe-Glu-X-X-Z (Figure 1), Leu was replaced by its hydrophobic analogues Nle, Ile, and Val in order to evaluate the influence of hydrophobicity on biological activity. 

All target peptides were synthesized using the standard protocol of solid-phase peptide synthesis (SPPS) according to Figure 5, without any specific problems during the synthesis.

### 3.2. Evaluation of the Analgesic Properties of the New Analogs

PPT is a technique for measuring the effectiveness of analgesics by observing the reaction of experimental animals to gradually increasing pressure on their hind paws. Pain sensitivity is modifiable by analgesics. Thus, the PPT of control animals can be compared to that of animals treated with substances with potential analgesic activity. The analgesic properties of all four newly synthesized analogs (**BB1**–**BB4**) vs. the parent compound (**BB11**) have been observed by means of PPT, differing in their duration and magnitude, and the obtained results are presented in Figure 2. The data reveals that **BB1**, the analog of the parent compound, where only C-terminal carboxyl function is transformed to amide one, led to a gradual increase in PPT during the total experiment time, i.e., 50 min. Animals that received **BB2**, the analog with the least sterically hindered side chain, or the parent compound **BB11** demonstrated constantly increased PPT for the total time of the evaluation. However, the non-modified parent compound, **BB11,** showed more prominent analgesia.

A prominent increase in PPT was observed on the 10th minute after **BB3** administration, where Leu residues are replaced by the structural analogue with shorter side chain Val, followed by a rapid decrease on the 20th minute, with values almost reaching the controls from the 40th minute on. A similar effect slope was registered for compound **BB4,** where Leu moieties are replaced by Ile. Although in **BB4** administration the increase occurred later, on the 20th minute, the analgesic activity persisted until the end of the experiment, showing even increasing values. 

Additional experiments for compounds **BB1,** a peptide having an analgesic activity that increases over time to become superior to the reference after 50 min, and the parent compound **BB11** were performed. Each compound was administered after Naloxone or AM251 pretreatment, and the data are presented in Figure 3. Naloxone and AM251 injections completely abolished the analgesia registered after **BB1** or **BB11** i.p. injections.

The obtained data showed that the analgesic activity of the newly synthesized analogs depends on both opioid and cannabinoid receptors, since antagonization of each of them completely abolishes the analgesic effect. Still, it seems that antagonization of opioid receptors abolishes analgesia from the first measurement (on the 10th minute) after **BB1** administration, while AM251 pretreatment led (even only) on the 10th minute to a higher level than the newly synthesized substance alone. Although such a difference could be attributed to the potential partial agonistic activity of AM251 (not officially recognized), no such differences were evaluated for **BB11,** where both naloxone and AM251 led to lower thresholds compared to the newly synthesized substance alone.

### 3.3. Hydrolytic Stability 

Hydrolytic stability is one of the most important features for applying new molecules in medicinal practice. Considering that one of the limiting factors for introducing the peptides in practice is their poor hydrolytic stability, the newly synthesized peptides were tested for their stability in three model systems that mimic different parts of the organism: pH 2 (stomach), pH 7.4 (blood plasma), and pH 9 (small intestine). The hydrolytic model systems were specifically designed to include the enzymes pepsin and trypsin at concentrations of 0.5 mg/mL and 0.1 mg/mL, respectively [34]. The tested compounds were used at a concentration of 1.0 mg/mL. The realized experiments revealed that all peptides were completely stable during a period of 24 h in the model medium. 

### 3.4. Lipophilicity 

The natural **BB11** peptide and its analogs have three hydrophobic amino acids and a polar one. They are short peptides (consisting of four amino acids), and the polarity of the groups at the N- and C-termini cannot be neglected—they contribute strongly to the generally low lipophilicity of the molecules. The amino acid composition is the source of the difference in log*p* values. Val is the most compact and hydrophobic residue. Nle and Ile have longer chains and show similar characteristics. Leu has a long chain and a branched end, giving it the most surface area for hydrophobic interactions, as we see in the natural **BB11**. Changing the hydroxyl group of the C-terminus with an amino one offers twice as many hydrogens for interactions with water molecules, making the **BB1** analog less lipophilic.

Regarding their activity, the most hydrophilic compound, **BB3**, has the highest initial activity, which then rapidly decreases as the peptide is probably hydrolyzed or otherwise removed from the receptors. Compounds **BB2** and **BB4** have almost identical log*p* values of 0.80 and 0.82, respectively. They have a peak at 20 min but then decrease as well. 

Lowering the lipophilicity of **BB1** compared to **BB11** has probably made it harder for the drug candidate to reach the target receptors, but it seems that once they are established in the appropriate place, their effect increases over time. 

## 4. Materials and Methods

### 4.1. Synthesis and Analysis of Targeted Peptides

#### 4.1.1. Materials 

All specifically protected amino acids Fmoc-L-Phe-OH, Fmoc-L-Glu(tBu)-OH, Fmoc-L-Leu-OH, Fmoc-L-Nle-OH, Fmoc-L-Ile-OH, and Fmoc-L-Val-OH, as well as Fmoc-Rink Amide MBHA and 2-Chlorotrityl chloride resin (2-CTC), activation agents *N*,*N*,*N′*,*N′*-tetramethyl-O-(1Hbenzotriazol-1-yl)uronium hexafluorophosphate (HBTU) and *N*,*N′*-diisopropylcarbodiimide (DIC), trifluoroacetic acid (TFA), scavenger triisopropylsilane (TIS), and base *N*,*N*-diisopropylethylamine (DIPEA) were purchased from Iris Biotech (Wunsiedel, Germany). The solvents *N*,*N′*-dimetylformamide (DMF) and dicholomethane (DCM) are obtained from Valerus (Sofia, Bulgaria), and 4-*N*,*N*-dimethylaminopyridine (DMAP) is from Sigma-Aldrich (Ansbach, Germany). All reagents and solvents were used without any preliminary treatment.

#### 4.1.2. Peptide Synthesis and Analyses

**Figure 6:**
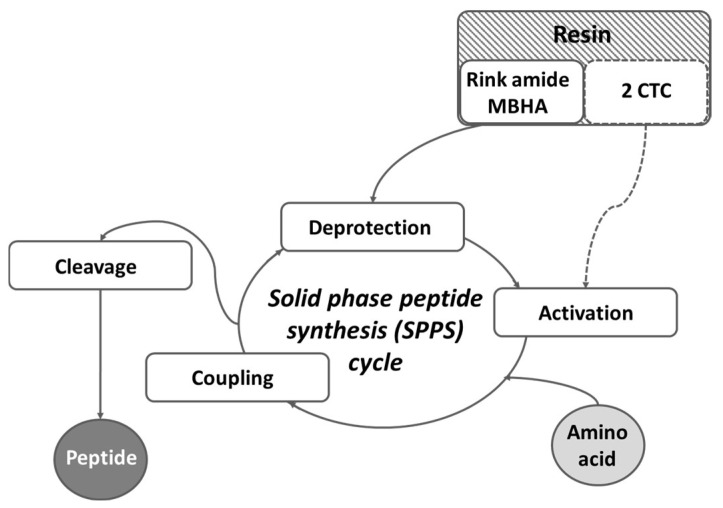
Schematic representation of the general SPPS cycle.

For the synthesis of targeted peptides, conventional solid-phase peptide synthesis (SPPS) by means of the Fmoc (9-fluorenylmethoxycarbonyl))/O*t*Bu strategy was used (Figure 6). 

Rink-amide MBHA or 2-CTC resins were used as solid-phase carriers, depending on the C-terminal modification. HBTU or DIC were used as condensation reagents and DIPEA or DMAP, respectively, as catalysts. The coupling reactions were performed using an amino acid/HBTU/DIPEA/resin molar ratio of 3/3/9/1 or an amino acid/DIC/resin molar ratio of 3/3/1 and a catalytic quantity of DMAP. The N^α^-Fmoc-group was deprotected at every step by treatment with a 20% piperidine solution in DMF. The coupling and deprotection reactions were checked by the standard Kaiser test. The cleavage of targeted peptides from the Rink Amide MBHA resin was performed using a mixture of 95% TFA, 2.5% TIS, and 2.5% distilled water. The cleavage of the parent FELL peptide from the 2-CTC resin was performed using a mixture of 50% TFA/50% distilled water: TIS (97.5:2.5 eq.). The peptides were obtained as oils in TFA and further precipitated in cold, dry diethyl ether. 

In brief, the synthesis of target compounds is realized using manual SPPS in the 20-milliliter glass reaction vessels purchased from Lipopharm.pl. The calculations are performed for 100 mg of final peptide, and the appropriate quantity of Rink-amide MBHA (load 0.63 mmol/g, 200–400 mesh) or 2-CTC (load 1.55 mmol/g, 100–200 mesh) resin is placed in the reaction vessel. Further, the first amino acid is attached to the resin using the method described in the Material and Methods, either directly for 2-CTC resin or after deprotection of the Fmoc-group on the Rink-amide MBHA resin (Figure 6). The process of synthesis of all target peptides repeats deprotection (20 min treatment with 20% piperidine in DMF) and condensation steps (4–6 h) until obtaining the aimed peptide sequence. Each step is monitored using the standard Kaiser test. The final step includes full deprotection of all amino acid residues from their protecting groups and removal from the resin using a cocktail depending on the type of resin. The yields of all targeted molecules are summarized in Table 1.

The peptide purity was monitored by HPLC, and their structures were proven by mass spectrometry (Supplementary Materials) using the following conditions: 

Shimadzu LC-MS/MS 8045 system (Shimadzu Corporation, Japan), Agilent Poroshell 120 (CA, USA), 100 mm × 4.6 mm column, mobile phase rate 0.30 mL/min, column temperature 40 °C. The following gradient elution was used: Mobile phase A: H_2_O (10% AcCN; 0.1% HCOOH); Mobile phase B: AcCN (5% H_2_O, 0.1% HCOOH). The gradient of the mobile phase starts with 80%A/20%B, passes through 5%A/95%B in 15 min, and returns to 80%A/20%B in 22 min.

The Mass Spectrometry detector was used in SCAN/ESI+ mode of ionization with 3 L/min of the nebulizing gas flow, 10 L/min of the heating and drying gas flow, a 350 °C interface temperature, a 200 °C DL temperature, and a 400 °C heat block temperature.

The optical rotation was measured on an automatic standard polarimeter, Polamat A, Carl Zeis, Jena (Anton Paar Opto Tec GmbH, Seelze, Germany), in dimethyl sulfoxide (DMSO) at c = 1. Melting temperatures were determined on a semi-automatic melting point meter M3000 by A. KRÜSS Optronic GmbH and are not corrected.

#### 4.1.3. Model Systems for Hydrolytic Stability Study

Three different pH values that mimic human pH in the stomach, blood plasma, and small intestine were selected for the investigation of the hydrolytic stability of newly synthesized compounds. Model solutions used for the determination of hydrolytic stability are prepared according to the European Pharmacopoeia, 6th Edition, as follows:

(i) A buffer with pH 2.0–6.57 g KCl is dissolved in water (CO_2_ free), and 119.0 mL of 0.1 mol/L HCl is added. A 0.5-gram aliquot of pepsin was added to the solution in order to obtain a 0.5 mg/mL final concentration. The obtained solution is diluted to 1000.0 mL with dH_2_O.
(ii) Buffer with pH 7.4–2.38 g Na_2_HPO_4_, 0.19 g KH_2_PO_4_, and 8.0 g NaCl are dissolved in dH_2_O. A 0.1-gram aliquot of trypsin was added to the solution in order to obtain a final concentration of 0.1 mg/mL. The obtained solution is diluted to 1000.0 mL with dH_2_O.
(iii) A buffer with pH 9.0–1000.0 mL of solution I is mixed with 420.0 mL of solution II. Solution I: 6.18 g H_3_BO_3_ is dissolved in 0.1 mol/L KCl, and it is completed to 1000.0 mL with the same solvent; Solution II: 0.1 mol/L NaOH. A 0.1-gram aliquot of trypsin was added to the solution in order to obtain a 0.1 mg/mL final concentration.

The chromatographic system used for determination of the hydrolytic stability included an HPLC model (Perkin-Elmer series 200, USA, Waltham, MA, USA), a Lichrospher RP-8 Non Endcpd column (pore size 5 µm, internal diameter 4.6 mm, and length 150 mm; Alltech, Lexington, KY, USA), and a UV detector (Perkin-Elmer series 200, USA) set at 274 nm, room temperature, and a flow rate of 0.70 mL/min with gradient elution: at the time 0.0 min 20%B; at the time 10 min 100% B; at the time 10 to 13 min 100% B; at the time 13 to 14 min 20% B; at the time 16.5 min 20% B. The mobile phase was prepared as follows: Solution A: Acetonitrile:Water:TFA—5:95:0.1; Solution B: Acetonitrile:Water:TFA—95:5:0.1 The injection volume was 20 μL.

#### 4.1.4. Lipophilicity Calculations

The modeled structures were geometrically optimized using the HyperChem software package [35]. The PM3 (Parameterized Model 3) level of theory was utilized for the calculations. PM3 is based on the concept of semi-empirical methods, which combine elements of quantum mechanics and empirical data to provide computationally efficient approximations of molecular properties. The theory incorporates a set of parameterized mathematical expressions to describe the behavior of electrons in molecules, making it less computationally demanding compared to ab initio quantum mechanical methods. The same software is used for the calculation of log*p*, a widely used approach in computational chemistry to estimate the partition coefficient of a compound between a non-polar organic solvent (*n*-octanol) and water. The partition coefficient is a measure of a compound’s hydrophobicity or lipophilicity, which is crucial in understanding its distribution and behavior in biological systems and drug design. The method is based on a fragment-based approach, where the molecule is divided into smaller fragments, or substructures. The contribution of each fragment to the overall log*p* value is determined based on a pre-established database of experimental log*p* values for known fragments. The sum of the fragment contributions provides an estimate of the overall log*p* value for the molecule.

### 4.2. In-Vivo Analysis

#### 4.2.1. Animals

The experiments were carried out on male Wistar rats (180–200 g) kept under normal conditions at ambient room temperature (22 °C). The animals were divided into five experimental groups, each including 8–10 animals, and a control group (n = 10). All experimental procedures were carried out between 10:00 a.m. and 1:00 p.m. after having been approved by the Research Ethics Commission of the Medical University—Sofia.

The control group received 0.2 mL of saline intraperitoneally injected (i.p.), while the animals from each one of the experimental groups received 0.2 mL (0.25 mg/kg, i.p.) of one of the five newly synthesized analogs dissolved in saline. 

#### 4.2.2. Nociceptive Test

##### Paw-Pressure Test (Randall-Selitto Test)

Changes in the mechanical nociceptive thresholds (PPT) of experimental animals were measured by an analgesimeter [36]. Pressure was applied to the hind paw, and the value (g) required to elicit a nociceptive response (i.e., a squeak or struggle) was taken as the mechanical nociceptive threshold. A cut-off value of 500 g was used to prevent damage to the paw.

Nociception was evaluated every 10 min from the 10th minute after the newly synthesized analog was administered until the 50th minute.

#### 4.2.3. Pretreatments

In order to estimate which type of receptor participates in the analgesic effects of the newly synthesized substances, pretreatments have been made with Naloxone—an antagonist with a high binding affinity for the MORs [37], clinically used to prevent fatal overdose consequences—and AM251—a CB1Rs’ antagonist [38].

#### 4.2.4. Data Analysis

The results were statistically assessed by one-way analysis of variance followed by a Newman-Keuls post-hoc comparison test. Values are represented as mean ± S.E.M. Values of *p* < 0.05 were considered to indicate statistical significance. 

## 5. Conclusions

Peptides, the “building blocks” of proteins, represent a special class of natural compounds. The development of peptide drugs (therapeutic peptides) is a hot topic in pharmaceutical research due to many of their advantages over conventional medical drugs. FELL is supposed to be the core anti-inflammatory motif in the CABS1 human protein [28]. Considering that inflammation in many cases is related to pain as well as that FELL is a structurally modified FEG/feG peptide that has different biological activities, an opioid activity of the FELL analogs can be presumed. In the study herein, new tetrapeptide analogs of FELL with a general formula Phe-Glu-X3-X^4^-Z, where X = Nle, Ile, or Val amino acid and Z = NH_2_ or COOH group, were synthesized by means of the standard SPPS, Fmoc/OtBu strategy in order to study the influence of structure and hydrophobicity on the analgesic activity of these peptides. The displayed activity of targeted molecules shows that all newly synthesized peptide analogs could become suitable starting substances for future syntheses in human’s battle against pain. The best activity is revealed for parent compound FELL (**BB11**) and its C-terminal amide analog (**BB1**), which means that Leu residues in positions 3 and 4 are the best choice as hydrophobic moieties. This conclusion is also proven by the in silico log*p* calculations. All newly synthesized molecules are completely stable in the model systems, which mimic the stomach, blood plasma, and small intestine for 24 h. 

Finally, the results showed that both opioid and cannabinoid receptors seem to be involved in the analgesic activity of the substances.

## Figures and Tables

**Table 1:** Analytical data for the prepared FELL analogs.

Code	Structure	Molecular Formula	Mm _exact,_ g/mol	[M + nH]^+^ _observed_	[M + Na]^+^_observed_	M.p., jjj °C	t_R_ Min	α_D_^20^, ^O^	Yield, %	Chromatographic Purity, %
**BB1**	H-Phe-Glu-Leu-Leu-NH_2_	C_26_H_41_N_5_O_6_	519.31	520.40	542.40	219 ± 221	5.317	−28	100	99
**BB2**	H-Phe-Glu-Nle-Nle-NH_2_	C_26_H_41_N_5_O_6_	519.31	520.45	542.40	221 ± 223	5.467	10	99	96
**BB3**	H-Phe-Glu-Val-Val-NH_2_	C_24_H_37_N_5_O_6_	491.27	492.40	514.35	223 ± 224	4.100	−10	83	96
**BB4**	H-Phe-Glu-Ile-Ile-NH_2_	C_26_H_40_N_4_O_7_	520.29	520.45	-	218 ± 211	4.950	−8	76	97
**BB11**	H-Phe-Glu-Leu-Leu-OH *	C_26_H_40_N_4_O_7_	520.29	521.39	543.40	219 ± 220	4.817	−8	46	98

* Peptide was first observed by Mathison et al. [31].

**Table 2:** Log*p* values calculated for FELL analogs.

Code	Log*p*
**BB1**	0.67
**BB2**	0.80
**BB3**	0.02
**BB4**	0.82
**BB11**	1.54

## Data Availability

Small amounts of the target compounds are available from the corresponding author.

## Supplementary Materials

The following supporting information can be downloaded at: https://www.mdpi.com/article/10.3390/ph16081183/s1, Figure S1. HPLC/MS profiles of targeted peptides.
