# Supplementary material for: Synthesis, In Silico Logp Study, and In Vitro Analgesic Activity of Analogs of Tetrapeptide FELL
Source: Pharmaceuticals (Basel). 2023 Aug 21;16(8):1183. doi: 10.3390/ph16081183 (PMC10458596; doi:10.3390/ph16081183)
Supplement: Supplementary file 1 [file pharmaceuticals-16-01183-s001.zip › pharmaceuticals-2490891-supplementary Figure S1.pdf]

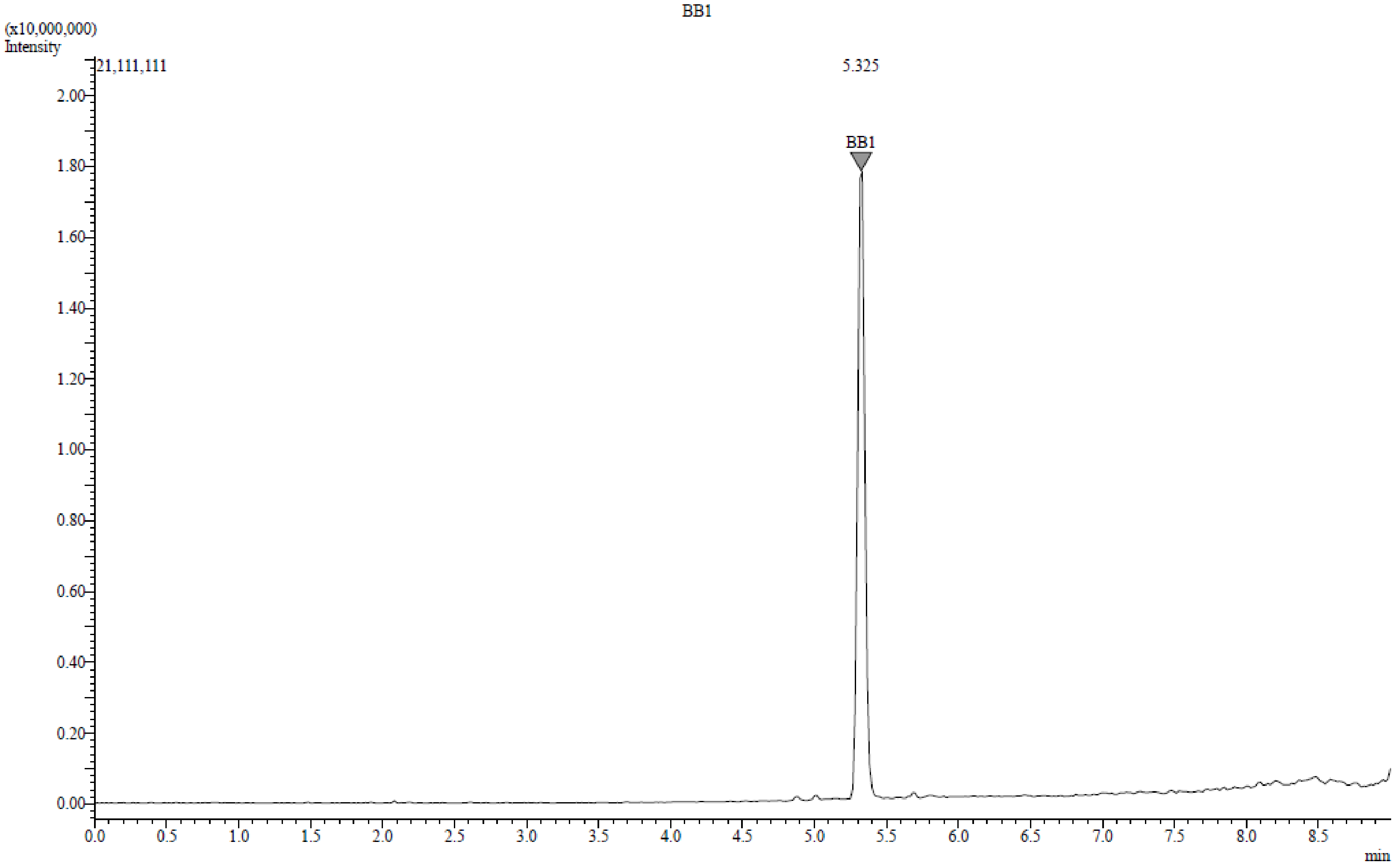

BB1
(x10,000,000)
Intensity
21,111,111
5.325
BB1
2.00
1.80
1.60
1.40
1.20
1.00
0.80
0.60
0.40
0.20
0.00
0.0 0.5 1.0 1.5 2.0 2.5 3.0 3.5 4.0 4.5 5.0 5.5 6.0 6.5 7.0 7.5 8.0 8.5
min

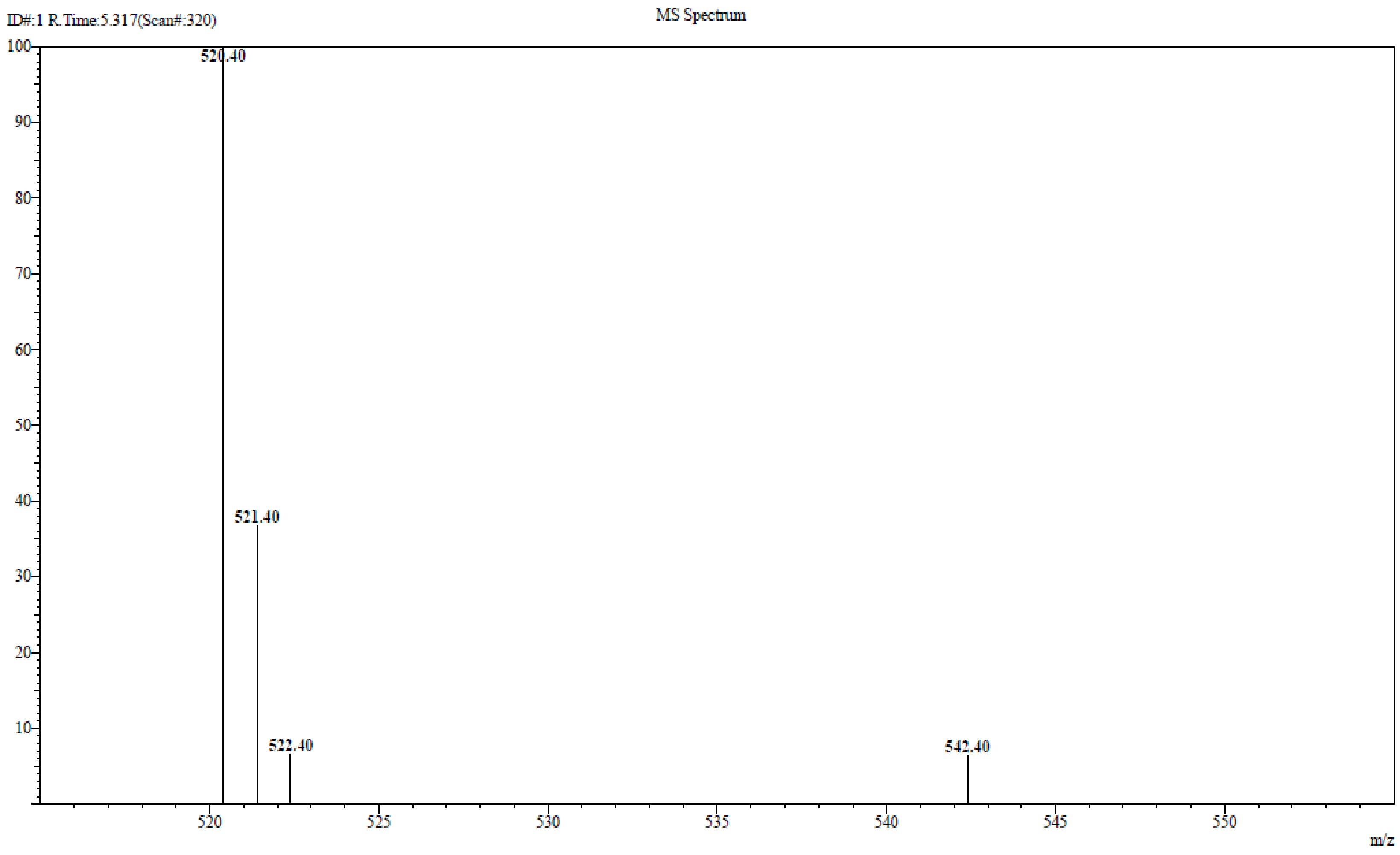
ID#:1 R.Time:5.317(Scan#:320)
MS Spectrum
520.40
521.40
522.40
542.40
100
90
80
70
60
50
40
30
20
10
520
525
530
535
540
545
550
m/z

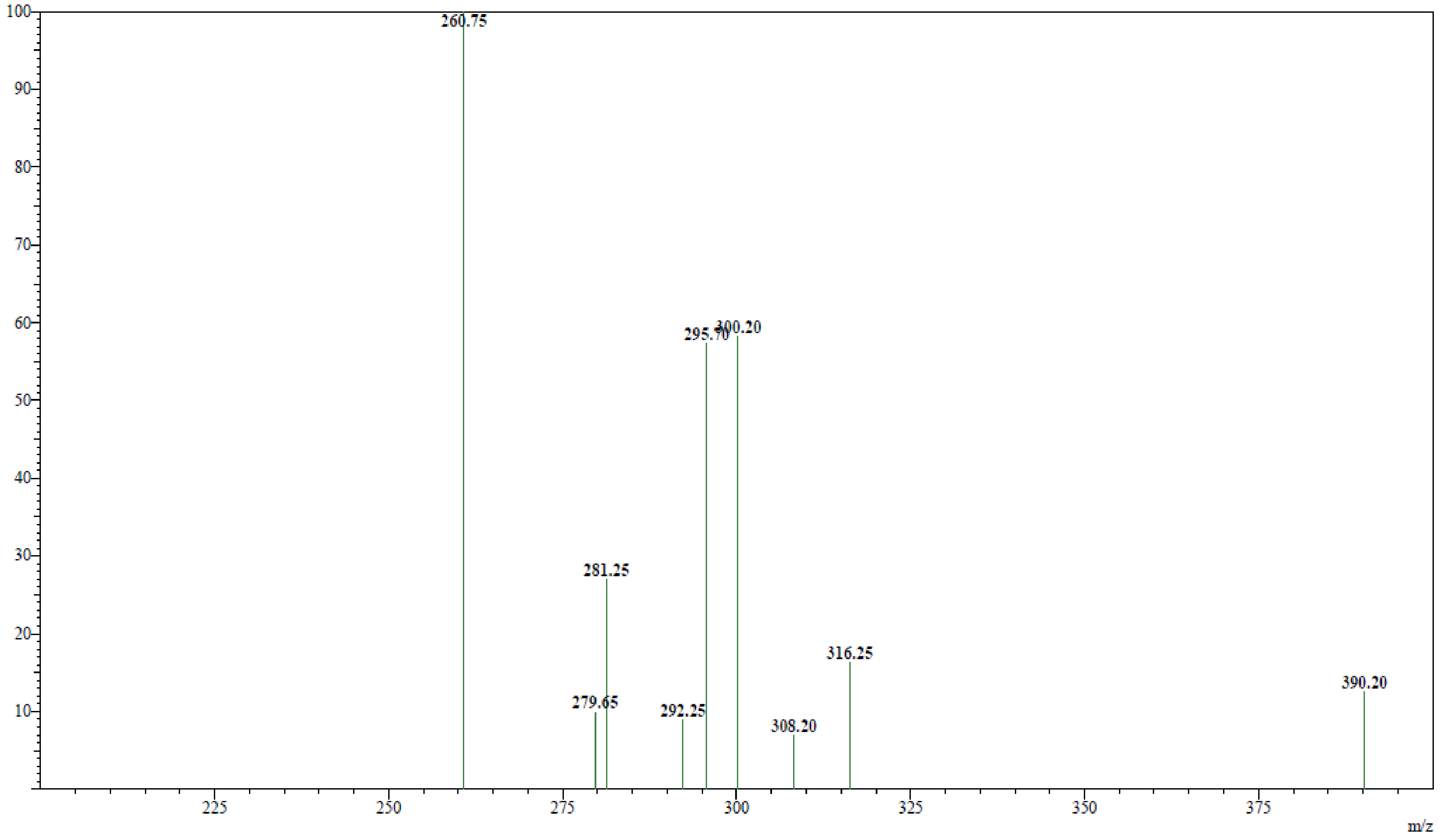
100
90
80
70
60
50
40
30
20
10
260.75
281.25
279.65
292.25
295.70
300.20
308.20
316.25
390.20
225
250
275
300
325
350
375
m/z

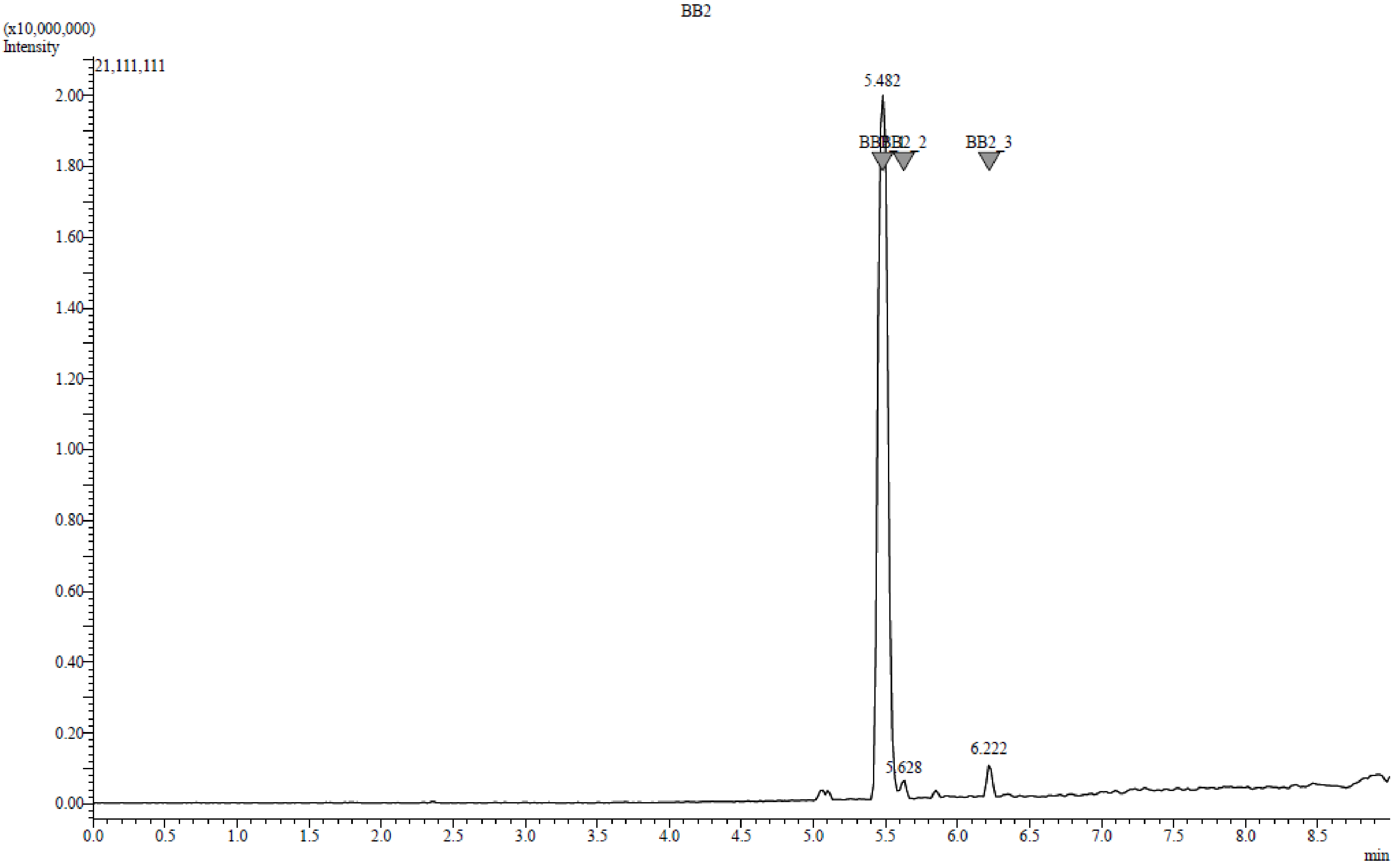

BB2
(x10,000,000)
Intensity
21,111,111
5.482
BB2
BB2_2
BB2_3
5.628
6.222
2.00
1.80
1.60
1.40
1.20
1.00
0.80
0.60
0.40
0.20
0.00
0.0
0.5
1.0
1.5
2.0
2.5
3.0
3.5
4.0
4.5
5.0
5.5
6.0
6.5
7.0
7.5
8.0
8.5
min

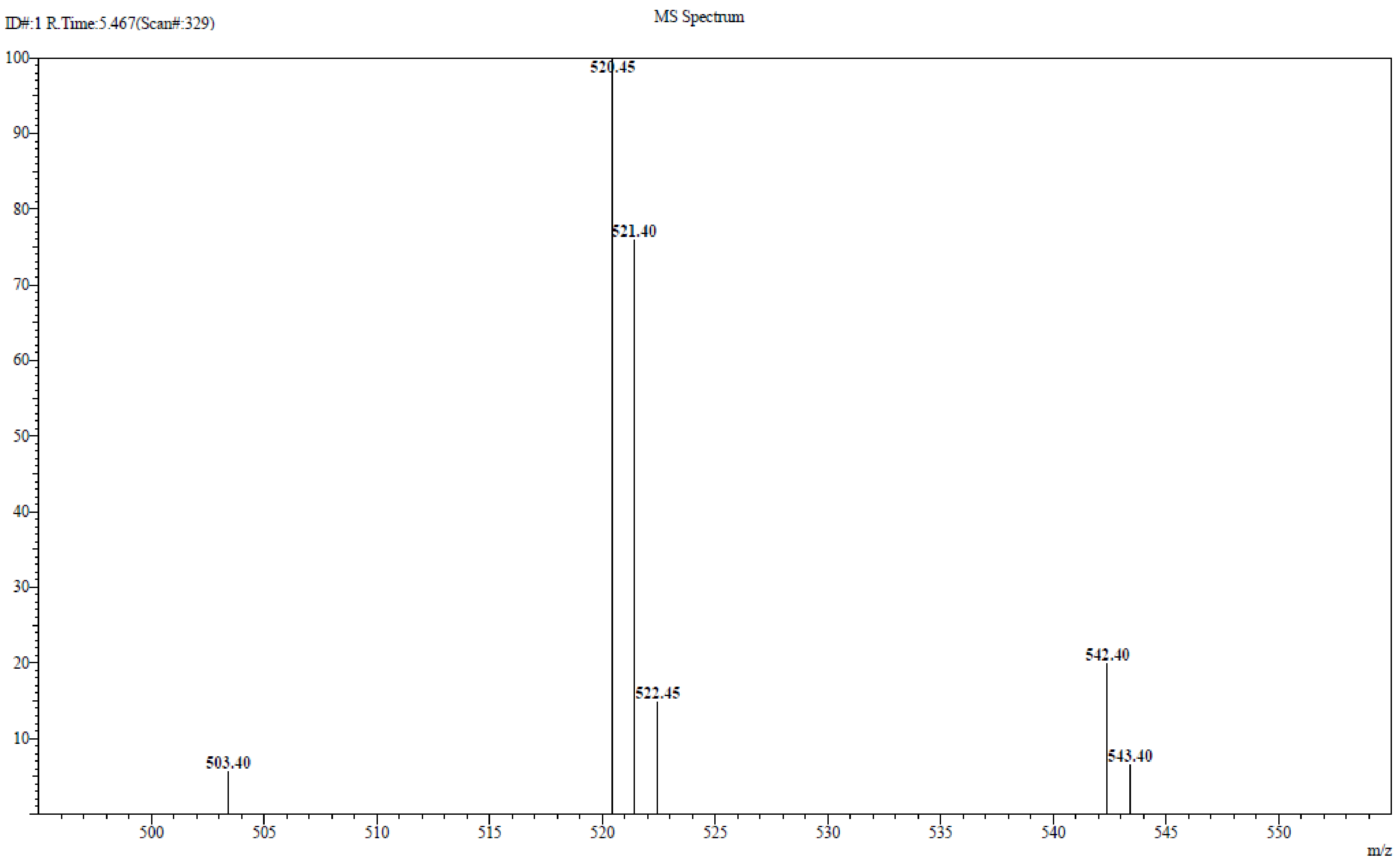

ID#:1 R.Time:5.467(Scan#:329)
MS Spectrum
520.45
521.40
522.45
503.40
542.40
543.40
m/z

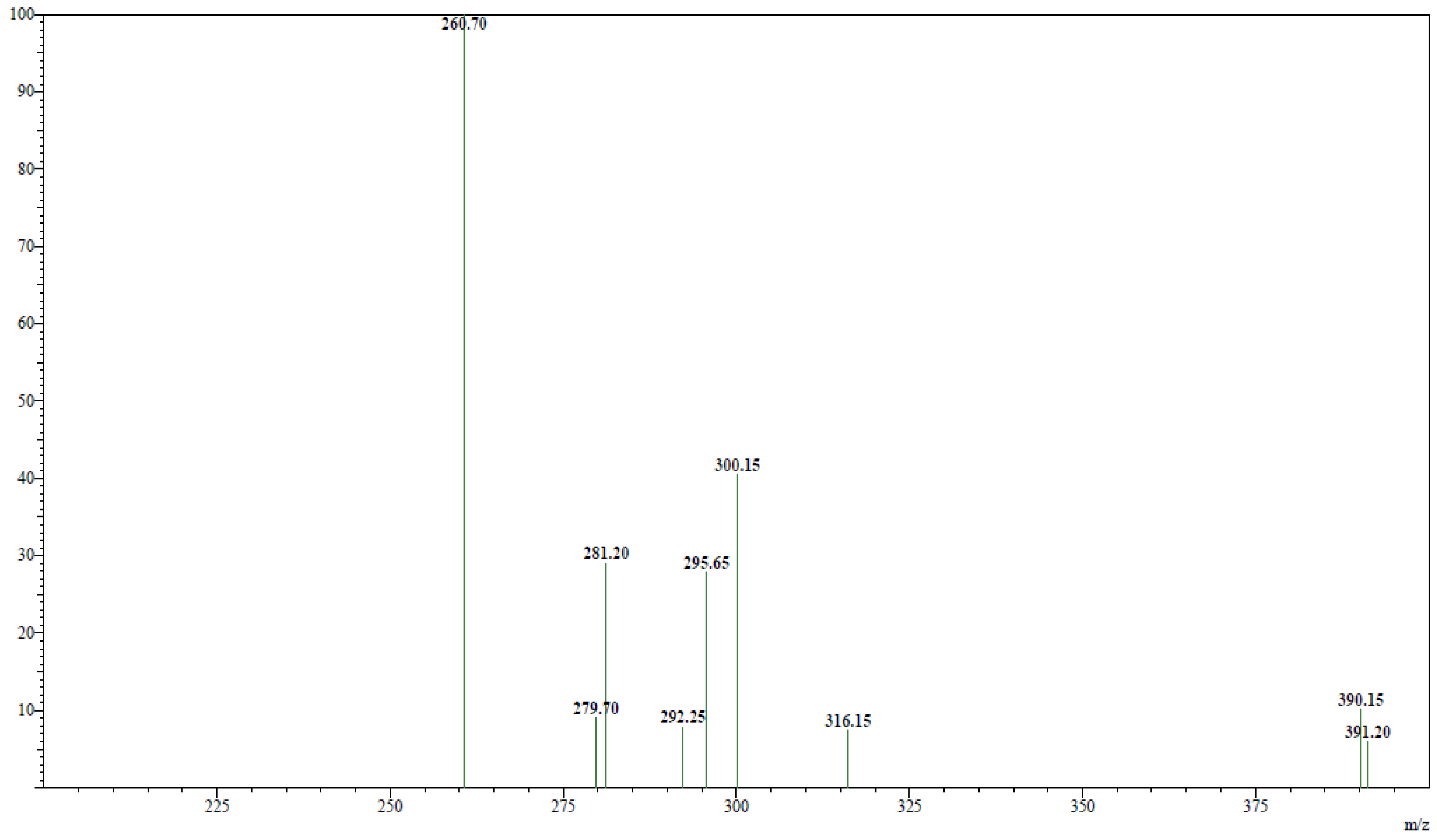

100
90
80
70
60
50
40
30
20
10
260.70
281.20
279.70
292.25
295.65
300.15
316.15
390.15
391.20
225
250
275
300
325
350
375
m/z

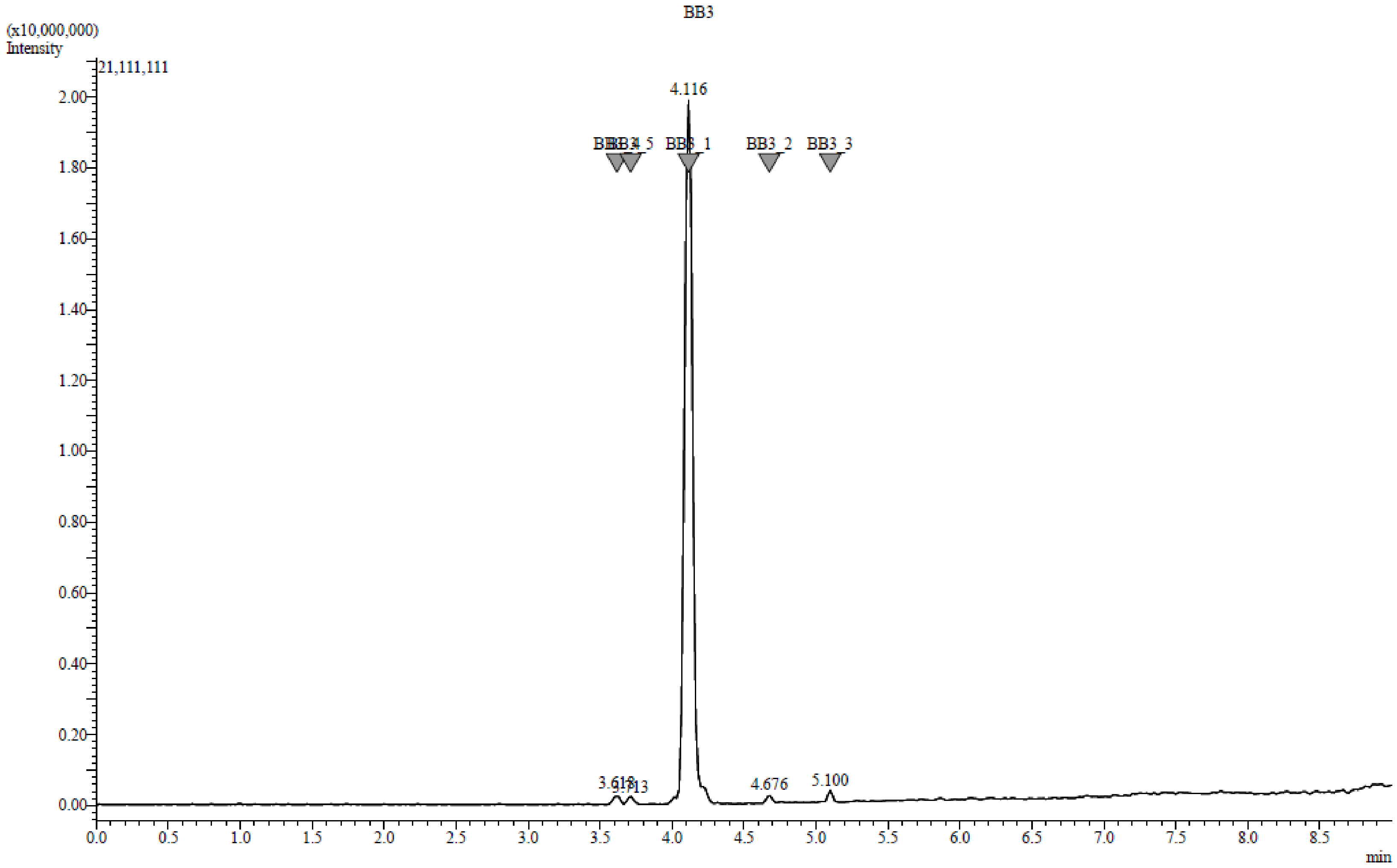

BB3
(x10,000,000)
Intensity
21,111,111
4.116
BB3_1
BB3_2
BB3_3
3.618
3.713
4.676
5.100
min

ID#:1 R.Time:4.100(Scan#:247) MS Spectrum

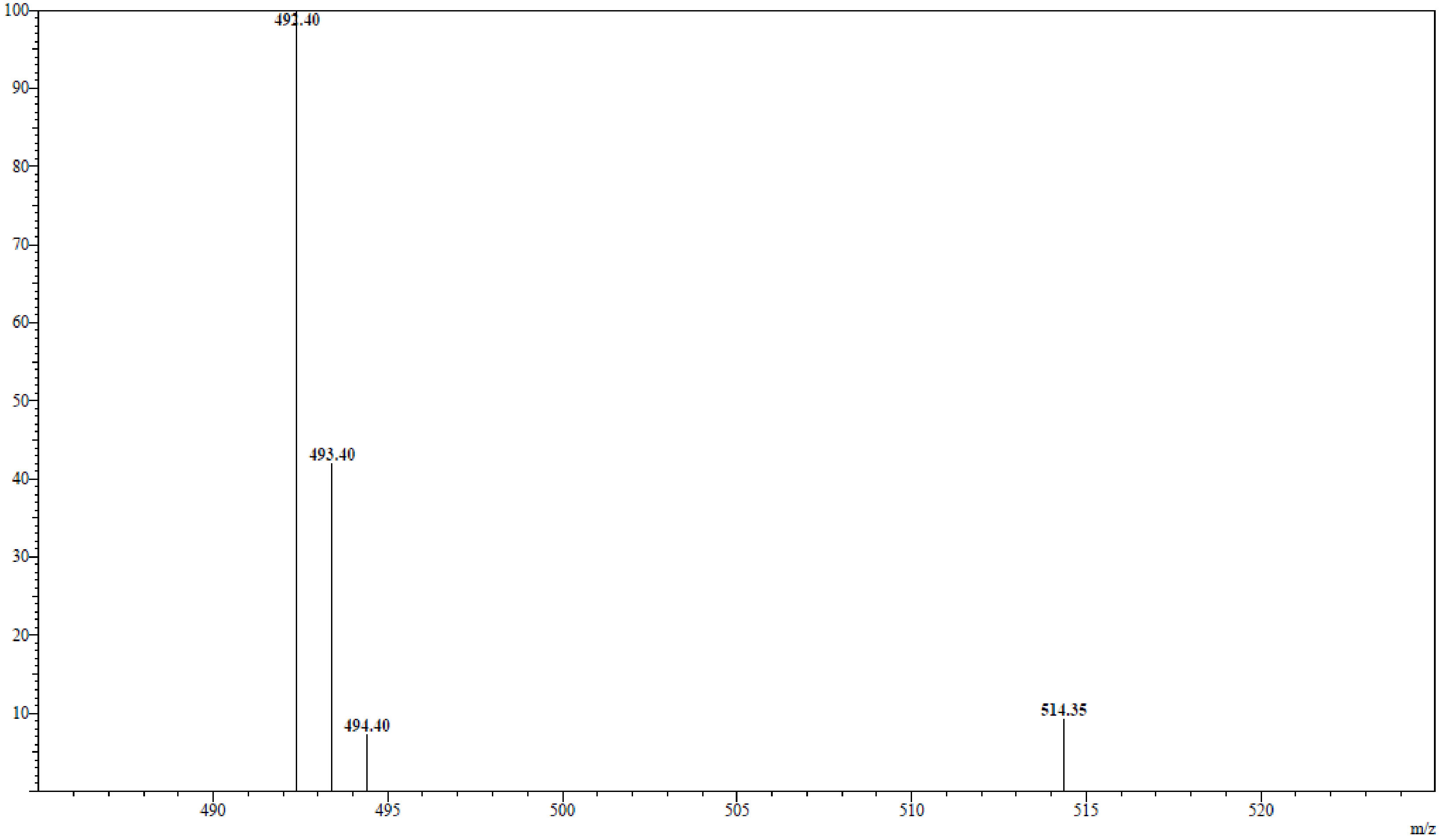

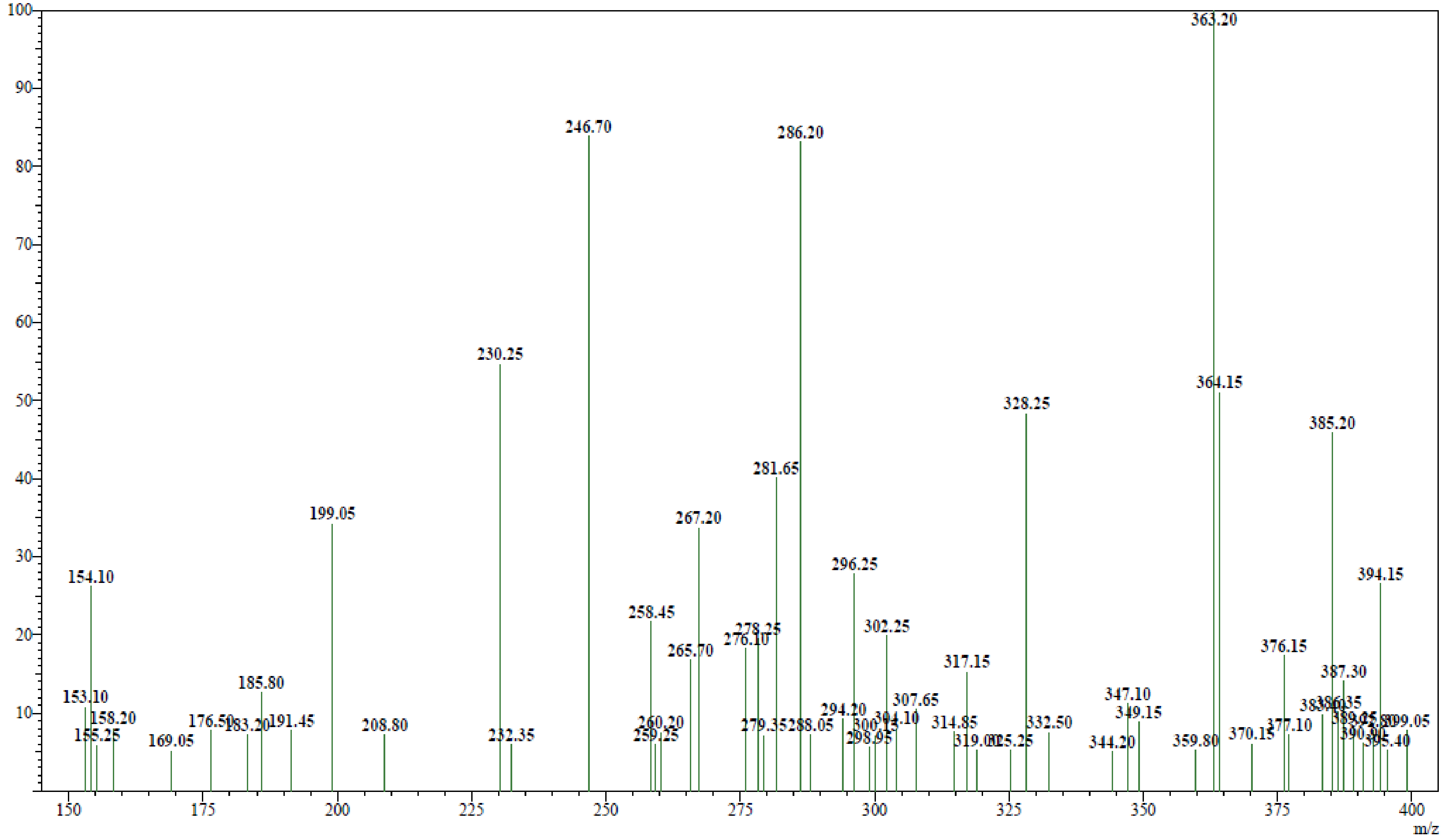
363.20
246.70
286.20
230.25
364.15
328.25
385.20
281.65
199.05
267.20
154.10
394.15
296.25
258.45
302.25
276.10
265.70
376.15
317.15
387.30
185.80
153.10
347.10
307.65
349.15
158.20
155.25
169.05
176.50
183.20
191.45
208.80
232.35
260.20
259.25
279.35
288.05
294.20
298.95
300.15
304.10
314.85
332.50
344.20
359.80
370.15
377.10
399.05
395.40
m/z

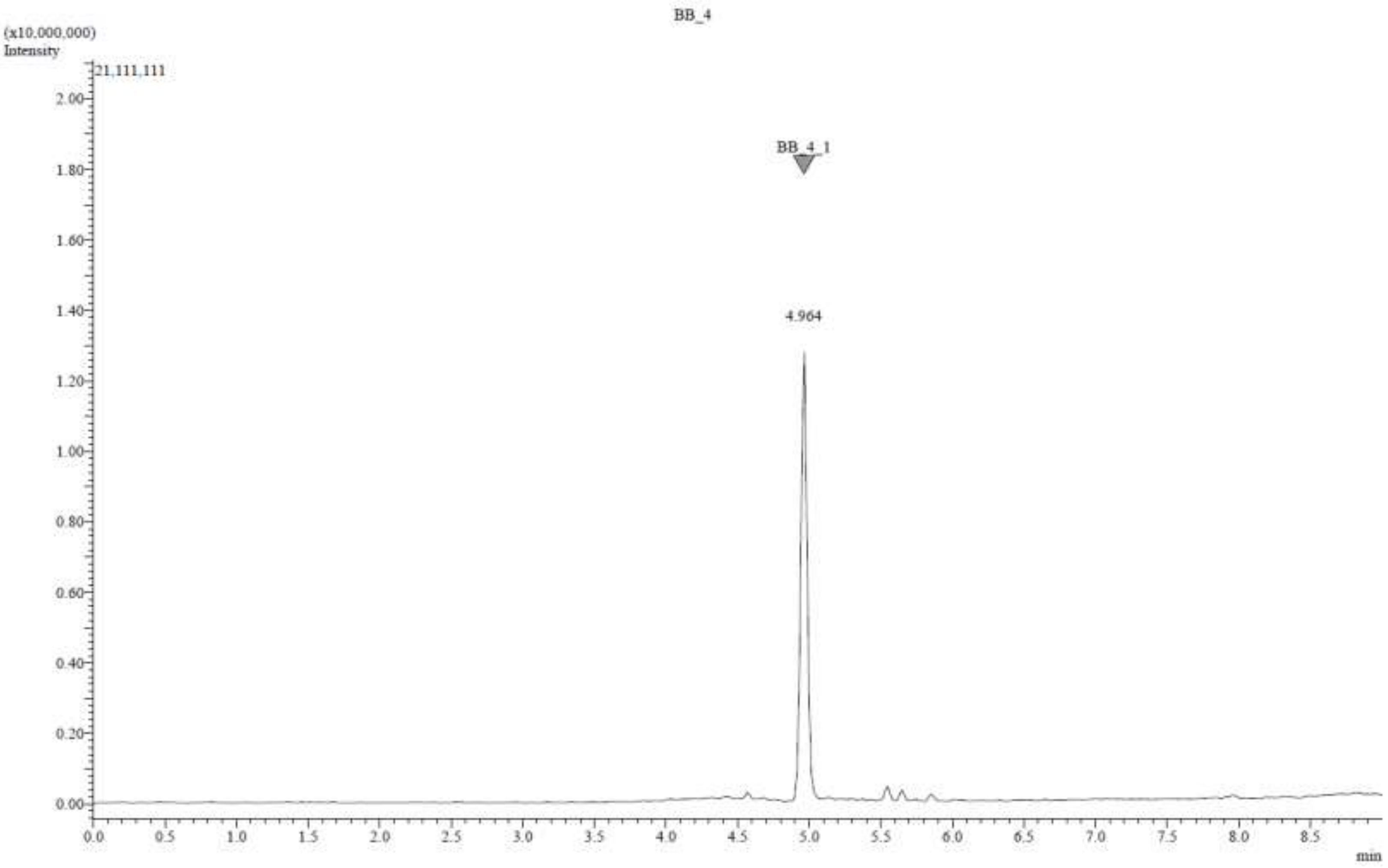

BB_4
(x10,000,000)
Intensity
21,111,111
2.00
1.80
1.60
1.40
1.20
1.00
0.80
0.60
0.40
0.20
0.00
BB_4_1
4.964
0.0
0.5
1.0
1.5
2.0
2.5
3.0
3.5
4.0
4.5
5.0
5.5
6.0
6.5
7.0
7.5
8.0
8.5
min

ID#:1 R.Time:4.950(Scan#:298)

MS Spectrum

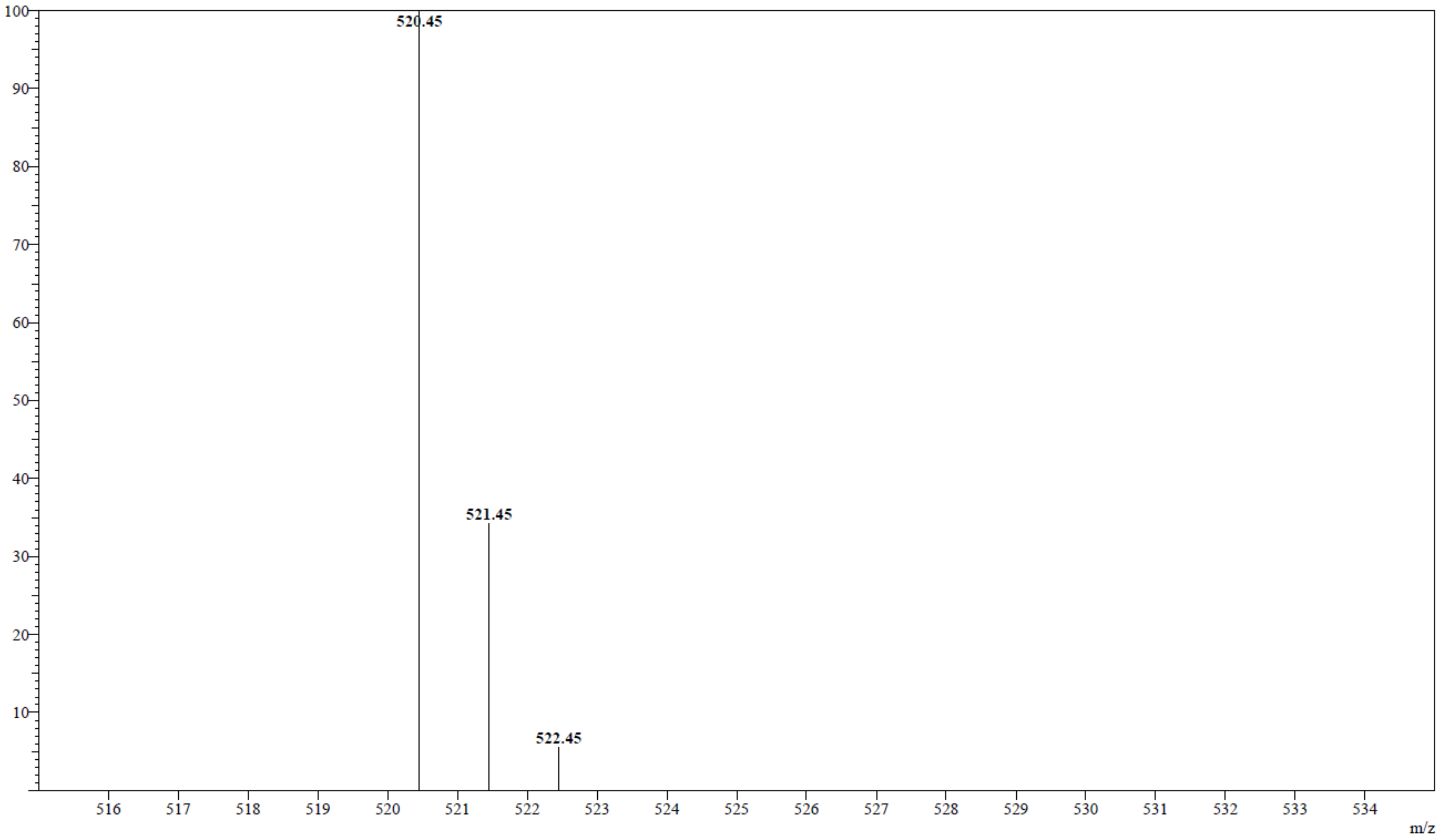

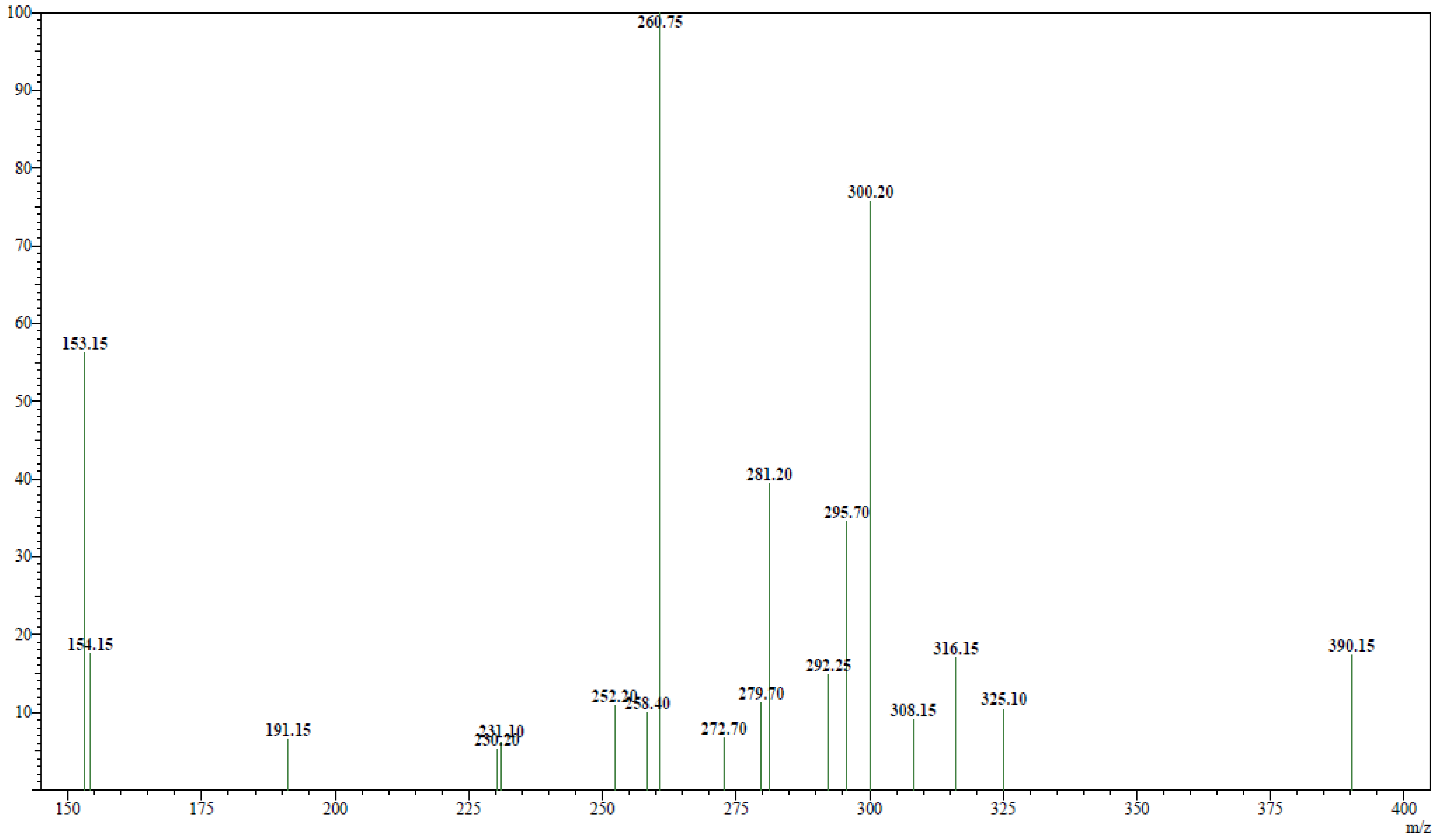

260.75
300.20
153.15
281.20
295.70
154.15
390.15
316.15
292.25
252.20
258.40
279.70
325.10
308.15
191.15
272.70
231.10
230.20
100
90
80
70
60
50
40
30
20
10
150
175
200
225
250
275
300
325
350
375
400
m/z

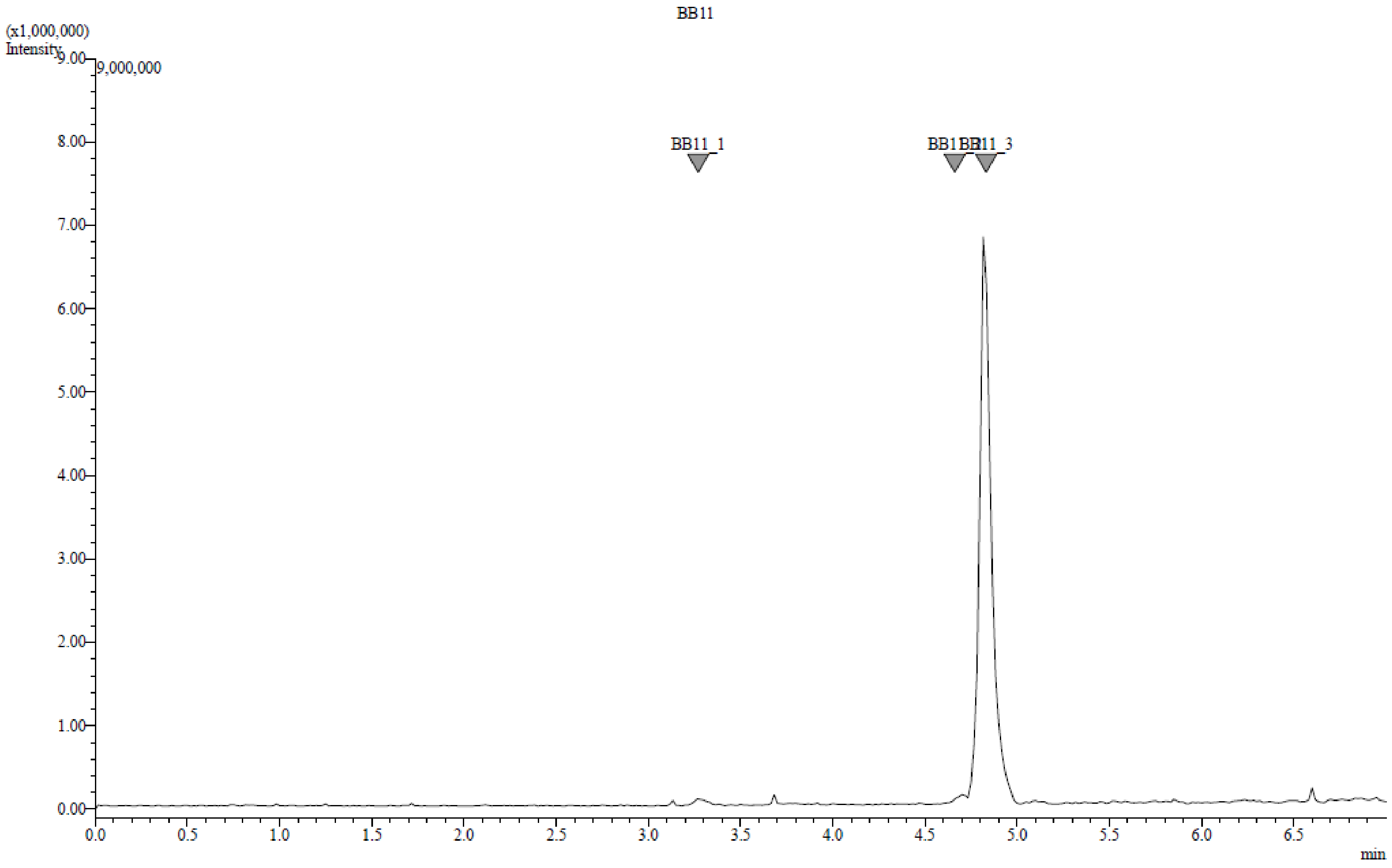
BB11
(x1,000,000)
Intensity
9,000,000
BB11_1
BB11_3
min

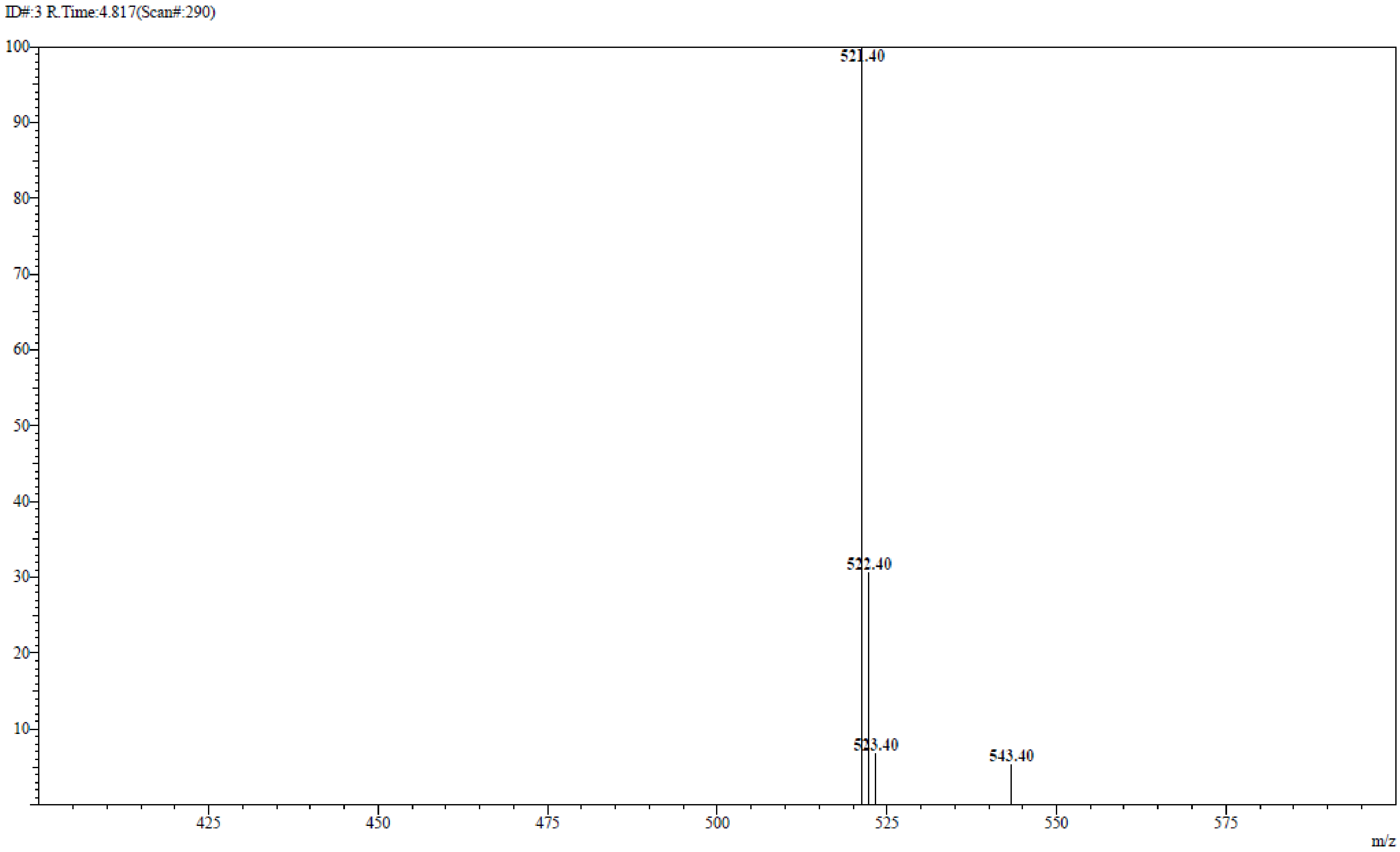
ID#:3 R.Time:4.817(Scan#:290)
521.40
522.40
523.40
543.40
100
90
80
70
60
50
40
30
20
10
425
450
475
500
525
550
575
m/z
